# Modern Sociology

**Published:** 1898-12-24

**Authors:** 


### MODERN SOCIOLOGY.

### THE LIQUOR TRADE IN AMERICA.

### II.—EMPLOYERS AND ALCOHOL.

THOUGH there are doubtless many employers of labour on this side who are affected in their choice of workmen by the fact that these are or are not abstainers, we do not think that the question has ever been made the subject of a Government inquiry here. Not so in the States, where the Commission appointed by the Government schedule the replies made by no less than 6,901 establishments, detailing their practice in regard to the taking on of the 1,745,923 employés in their service. The employers include individuals or companies engaged in agriculture, manufactures, mining and quarrying, transportation, and retail trade. The habits and opinions of these different employers are naturally varied. With 1,613 the report is that the habits of the prospective employés regarding drinking are not taken into consideration; but the large majority, viz., 5,363, take means to discover what a man's habits are. The means employed to this end are varied in the extreme. One farmer reports that he offers a man drink, and, if he takes it, refuses to employ him. We are glad there is only one of this kind. It seems to us a device to entrap a man, and shows a lack of that fair and open dealing between man and man which is nowhere more required than in the relation of master and man. Moreover, it would soon be ineffective. Intending workers would soon learn of Mr. ——'s notion of testing a man, and those most given to drink would the most ostentatiously refuse it at his hands. More satisfactory, because more honourable, and appealing to a higher instinct in the worker, is the habit of eight transportation companies, which demand that their employés shall sign a bond that they will not partake of intoxicants. Here there is fair and open dealing, the employé knows what he is pledged to do, and it is at his own risk if he fails to keep his promise.

This plan may, and probably does take account of a man's appearance and what is known of his previous record, though this does not appear in the answer. Of course these points affect many employers, especially in small places, where such knowledge is easily obtainable. Personal knowledge, added to appearance, reputation, recommendations, and inquiries, especially from former employers, are relied on in many cases; though it appears that many employers rely only on the applicant's appearance to judge whether or not he is sufficiently sober to suit them. No fewer than 433 employers report that this is the sole method they use to discover if the applicant for a situation is a temperate man or not; 360 add to this the replies that the candidate gives to their questions; while 410 rely on questioning alone. Recommendations of former employers naturally carry weight; but the number of employers who rely on these form but a small proportion of the whole. Simple inquiry, not necessarily from former masters, but from other (presumably reliable) acquaintances of the applicant, is the plan resorted to in most cases, the number of employers who take this means standing at 901.

Besides those who object to their employés taking intoxicants at any time, whether on or off duty, there are a number of employers who insist merely that while actually at work they should abstain from liquor, and also those who, while they do not lay any restraint on the majority of their employés, insist on those in responsible positions refraining from drink. Thus, in all departments of industry there are a good many firms who demand abstinence from engineers, managers, and watchmen. In agriculture stress is laid on teamsters being non-drinkers, for fear of carelessness and cruelty in the handling of cattle on the part of intoxicated men. In mining and quarrying the restriction is applied chiefly to handlers of explosives, and to electricians and the like, any blunder on whose part might lead to great injury to their fellow-workers, not to speak of damage to property. In transportation almost all branches of the service are, in some companies, forbidden to have anything to do with alcohol: trainmen, motormen, conductors, telegraph operators, electricians, switchmen, and pilots. The responsibility attached to these duties is so clear that the restriction needs no comment. But it may be doubted if forbidding men to drink merely when on duty is really of great service. If a man is half drunk, or stupid with drink which he has not fully slept off, he is as likely to be guilty of some fatal blunder as if he drank when absolutely at work. But doubtless those employers who forbid drinking when at work also take into account the condition in which a man who is not an abstainer is likely to commence his duties.

There is a general impression that persons employed on night duty are more given to excess in the use of stimulants than those who work by day. Night work being an unnatural form of labour, however necessary it may be in certain cases, a resort to stimulants to maintain an artificial energy during the time the work goes on, seems to be very probable. Yet this view does not seem to be completely borne out by American statistics. The Commission canvassed 1,659 employers on this subject; of these 53 did not answer. Of those who replied 141 said that those of their employés who had night duty to perform were more addicted to liquor than others; but 1,460 employers reported just the opposite, and expressed the conviction that their night workers had no more craving for intoxicants than the rest. Of course this leaves one free to deduce either that night work is less of a hardship that would drive men to liquor than is generally supposed, or, if one is cynical, that in these 1,460 workshops the day workers are not so conspicuously sober that they compare favourably with those who are employed by night. Over-work and exposure to severe weather are other excuses often given for over-indulgence in stimulants. But here we find the same thing; the number of employers whose workers are engaged overtime is, indeed, so small that one can hardly form a reliable judgment from it. Of the establishments canvassed, 6,379 had no employés who worked overtime, and of the remaining 553 whose opinions were asked 23 did not reply; but of the 535 remaining, only 99 said that those who did overtime work were more dissipated than their fellows; while 436 reported that there was no difference. Exposure to weather seems to have more influence than overwork. The number of establishments reporting that those of their employés who were thus exposed were addicted to intoxicants is 381; but, on the other hand, no less than 1,619 establishments say that this makes no difference. The same holds good of casual workers. Nearly 2,000 establishments who employed casual hands were canvassed on this point. Of these 313 gave no reply. Of the remainder 391 thought that these irregularly employed hands were less sober than others, but 1,214 reported the reverse. If one may draw an inference it seems to be this: that extra strain or irregularity of employment will afford a man who inclines to drink an excuse for drinking more; but the man who is by nature or principle sober, is sober no matter what are the conditions of his work.

